# Circadian and Social Cues Regulate Ion Channel Trafficking

**DOI:** 10.1371/journal.pbio.1000203

**Published:** 2009-09-29

**Authors:** Michael R. Markham, M. Lynne McAnelly, Philip K. Stoddard, Harold H. Zakon

**Affiliations:** 1 Section of Neurobiology, Patterson Laboratory, The University of Texas at Austin, Austin, Texas, United States of America; 2 Institute for Neuroscience, Patterson Laboratory, The University of Texas at Austin, Austin, Texas, United States of America; 3 Department of Biological Sciences, Florida International University, Miami, Florida, United States of America; University of Ottawa, Canada

## Abstract

Electric fish generate and sense electric fields for navigation and communication. These signals can be energetically costly to produce and can attract electroreceptive predators. To minimize costs, some nocturnally active electric fish rapidly boost the power of their signals only at times of high social activity, either as night approaches or in response to social encounters. Here we show that the gymnotiform electric fish *Sternopygus macrurus* rapidly boosts signal amplitude by 40% at night and during social encounters. *S. macrurus* increases signal magnitude through the rapid and selective trafficking of voltage-gated sodium channels into the excitable membranes of its electrogenic cells, a process under the control of pituitary peptide hormones and intracellular second-messenger pathways. *S. macrurus* thus maintains a circadian rhythm in signal amplitude and adapts within minutes to environmental events by increasing signal amplitude through the rapid trafficking of ion channels, a process that directly modifies an ongoing behavior in real time.

## Abstract 2

Electric fish strengthen their communication signals nightly and during social encounters by rapidly trafficking ion channels into cell membranes, demonstrating a direct relationship between environmental stimuli, channel trafficking, and behavior.

## Author Summary

Excitable cells, such as neurons and muscle cells, control behavior by generating action potentials, electrical signals that propagate along the cell membrane. Action potentials are generated when the cell allows charged molecules (ions) such as sodium and potassium to move across the membrane through specialized proteins called ion channels. By changing the number of ion channels in the plasma membrane, excitable cells can rapidly remodel their functional characteristics, potentially causing changes in behavior. To gain an understanding of how environmental events cause the remodeling of excitable cell membranes and the resulting behavioral adaptations, we studied the electric communication/navigation signals of an electric fish, *Sternopygus macrurus*. High amplitude signals facilitate communication and electrolocation, but are energetically costly and more detectable by those predators that can detect electrical signals. We found that *Sternopygus* increase signal amplitude at night, when they are active, and increase signal amplitude rapidly during social encounters. Electrocytes, the cells that produce the signal, rapidly boost the signal amplitude when they allow more sodium to cross the cell membrane, thereby generating larger action potentials. To increase sodium currents during the action potential, electrocytes rapidly insert additional sodium channels into the cell membrane in response to hormones released into circulation by the pituitary. By adding new ion channels to the electrocyte membrane only during periods of activity or social encounters and removing these channels during inactive periods, these animals can save energy and reduce predation risks associated with communication.

## Introduction

Electric fish generate electric organ discharges (EODs) by the simultaneous action potentials (APs) of excitable cells (electrocytes) in the electric organ. These nocturnally active fish detect distortions of the EOD to locate objects around themselves and communicate by broadcasting their EODs to conspecifics. Larger amplitude EODs allow an individual to broadcast its signal further to sense its surroundings and communicate with conspecifics. On the other hand, EODs can be energetically costly to produce [1] and some EODs may attract electroreceptive predators [2],[3],[4], making high-amplitude EODs particularly costly and dangerous. Increasing EOD amplitude only during periods of feeding and social encounters might be a way for these animals to balance the benefits of high-amplitude EODs against the energetic costs and predation risks associated with high signal amplitudes.

**Figure 1:**
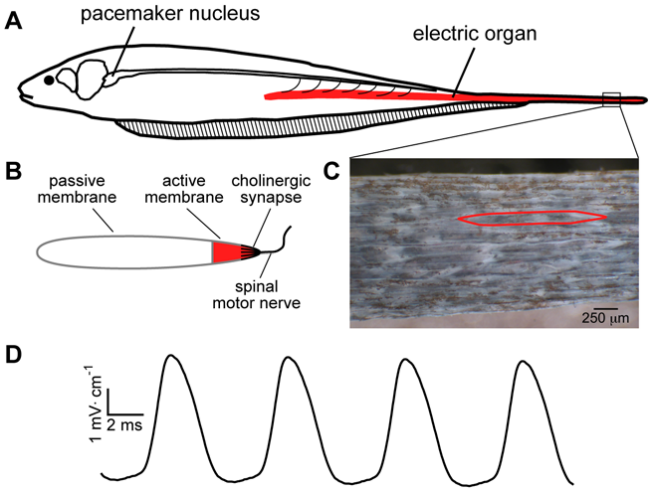
Generation of the EOD. (A) The EOD is produced by the coordinated APs of the electric organ cells, called electrocytes. A medullary pacemaker nucleus controls the electrocyte APs via spinal electromotor neurons which innervate the electrocytes. (B) Electrocytes are innervated on the posterior end of the cell, where the spinal nerve forms a large cholinergic synapse. The electrically excitable region of the cell membrane, populated by Na^+^ and K^+^ channels, is localized to the posterior most region of the cell, extending approximately 150 µm toward the anterior of the cell. The remainder of the cell membrane is electrically passive. APs in the electrocytes cause current to move along the rostral-caudal body axis and out into the surrounding water. (C) A section of electric organ from the tail, with skin removed to expose the electrocytes, which are densely packed within the electric organ. A single electrocyte is outlined in red. (D) The EOD waveform recorded from *S. macrurus* is a sinusoidal wave emitted at a steady frequency by each fish. The EOD frequency among fish has a range of approximately 70 to 150 Hz.

Two broad classes of electric fish are defined by the rate and regularity of their EODs. Pulse-type fish emit EODs at low-rate irregular intervals and can accelerate or decelerate their EOD rates, potentially saving energy and reducing predation risk by slowing their discharge rate. Wave-type fish constantly emit EODS at a high frequency, limiting their ability to regulate energy expenditure by reducing the discharge rate. One pulse-type species, *Brachyhypopomus pinnicaudatus*, not only modulates the discharge rate but increases EOD amplitude up to 25% during the night when socially active, decreasing EOD amplitude to baseline during the day [5]. Waveform modulation is mediated by circulating melanocortin peptides such as adrenocorticotropic hormone (ACTH) [6],[7]. We chose to study the cellular mechanisms of ion channel regulation in the wave-type gymnotiform fish *Sternopygus macrurus* (Figure 1) because this fish emits a simple EOD waveform and the ion currents responsible for generating its EOD are known [8], enabling us to determine the exact ionic mechanisms of EOD amplitude regulation and estimate their energetic costs. Moreover, we reasoned that EOD modulation would be greater in a wave fish because modulation of EOD amplitude is the only means for these animals to reduce the energetic cost of the EOD.

We hypothesized that changes in EOD amplitude are mediated by trafficking additional ion channels into the electrocyte's active membranes. Trafficking pre-synthesized molecules into the plasma membranes of excitable cells allows animals to remodel excitable cells within minutes [9]. Rapid trafficking of voltage-dependent ion channels has been demonstrated in vitro for cardiac and neuronal cells [9],[10],[11],[12],[13], but it is less clear how such processes contribute to adaptive behavior of the whole animal. Here we demonstrate in *S. macrurus* that rapid, controllable trafficking of ion channels is responsive to environmental cues and modifies electrical signaling behavior in real time, allowing *S. macrurus* to increase EOD amplitude during periods of activity or social interaction.

## Results

### Diurnal Fluctuations in *Sternopygus* EOD Amplitude Are Large

**Figure 2:**
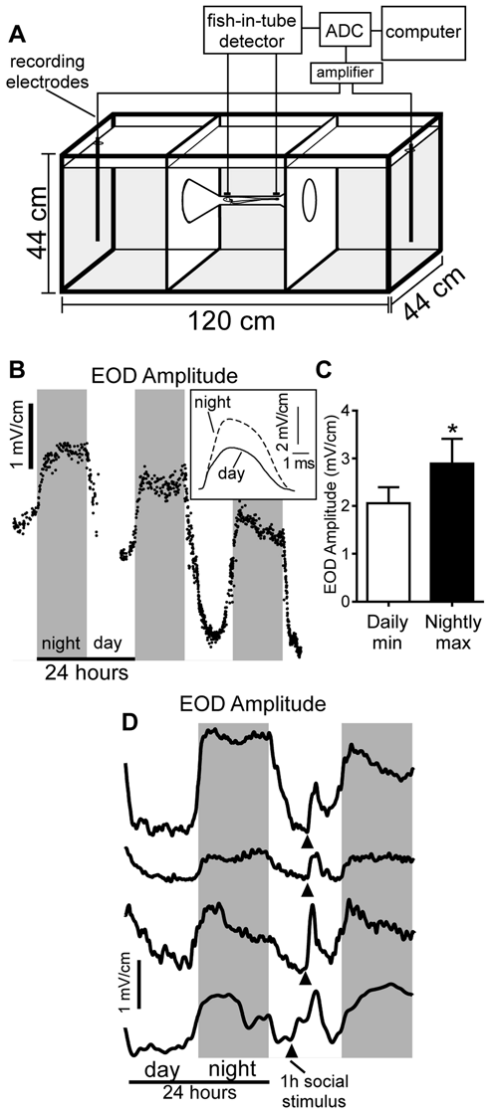
Circadian and social cues increase EOD amplitude. (A) Experimental tank used to record calibrated EODs of free-swimming fish. EODs were digitized from nichrome recording electrodes at the ends of the tank only when circuitry detected that the fish was centered within an unglazed ceramic tube, equidistant from the recording electrodes at the ends of the tank. ADC, analog-to-digital-converter. (B) EOD amplitudes of a representative fish recorded approximately every 60 s over 3 d. Signal amplitude shows a clear day-night rhythm increasing to maximum during lights-out and decreasing to a minimum at midday. Inset: superimposed EOD waveforms taken from the same fish at nighttime maximum and daytime minimum. (C) EOD amplitudes were significantly higher at nighttime peak than at daytime minimum (*n* = 8, *t* = 3.91, df = 7, *p*<0.01). Bars show means, and error bars indicate SEM. (D) Adding a second fish into the center compartment for 1 h at midday (arrowheads) caused transient increases in EOD amplitudes of four fish. All voltages are referenced to a point 10 cm from the center of a 5 cm calibration dipole [38].

We recorded calibrated EODs noninvasively from free-swimming *S. macrurus* and found that EOD amplitude varies diurnally (Figure 2B and 2C), increasing by 39%±5.5% from the daily minimum to the nighttime maximum. Social encounters with conspecifics that allowed electrical but not physical interaction produced similar increases in EOD amplitude (Figure 2D). As predicted, this wave species shows an approximately 2-fold larger modulation in its EOD amplitude than the previously studied pulse gymnotiform species, *Brachyhypomus pinnicaudatus*.

**Figure 3:**
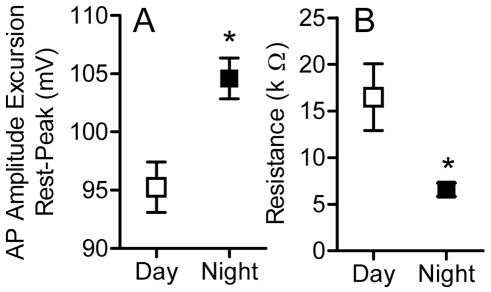
Electrocyte AP amplitude is higher at night. (A) AP amplitude (measured from resting potential to AP peak) is higher in electrocytes harvested at night than in those taken during the day (*t* = 3.39, df = 48, *p*<0.01). (B) Night-harvested electrocytes have lower input resistances than those sampled during the day (*t* = 2.676, df = 43, *p*<0.05).

Because EOD pulse characteristics are shaped directly by the electrocyte APs [14], we hypothesized that increases in EOD amplitude induced by encroaching darkness or social interaction reflect increases in the amplitude of APs in individual electrocytes. Using standard two-electrode current-clamp procedures, we recorded APs from electrocytes in the isolated electric organ harvested during daytime (11:00–13:30 h) or nighttime (20:00–23:30 h). In three fish we harvested the electric organ first during the day, and then at least 36 h later took a second piece of electric organ at night. Three additional fish were sampled in reverse order. With each piece of electric organ we recorded APs from at least four electrocytes within 45 min of tissue harvest. Electrocyte AP amplitude was higher and input resistance was lower in electrocytes taken from fish at night (Figure 3).

**Figure 4:**
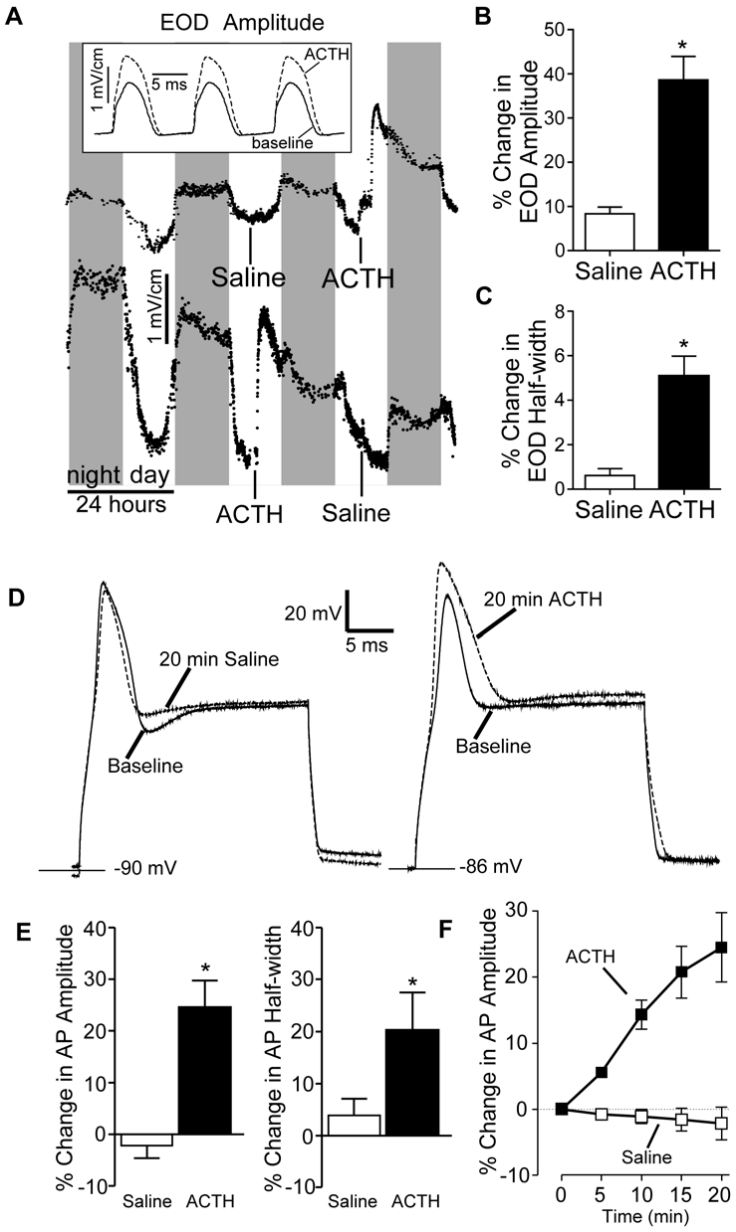
ACTH increases EOD amplitude in vivo and electrocyte AP amplitude in vitro. (A) Injections of ACTH increased EOD amplitude whereas saline injections have little or no effect. Plots show EODs recorded from two fish that received counterbalanced injections of saline or ACTH at midday. Inset: superimposed representative EOD waveforms taken at baseline and 1 h after ACTH injection. (B, C) Percentage increase in EOD amplitude and half width following injections of saline or ACTH injections. Bars show means, and error bars indicate SEM. (D) Representative traces of APs recorded at baseline and after 20 min exposure to saline (control) or ACTH. (E) In cells exposed to ACTH, AP amplitude and half width increased compared to saline controls. Both experimental and control cells showed slight but similar increases in input resistance (unpublished data). Asterisks indicate conditions different from saline control (unpaired *t* test, *p*<0.05). (F) Time course of ACTH-induced increase in AP amplitude.

Melanocortin peptide hormones act directly on the electric organ to modulate the EOD waveform in the pulse-type gymnotiform *B. pinnicaudatus* [7] and plasma levels of the pituitary melanocortin peptide ACTH follow a diurnal rhythm in teleost fish [15], suggesting that melanocortin peptides underlie circadian and social modulations of EOD amplitude in *S. macrurus*. We attempted without success to determine plasma ACTH levels by radioimmunoassay, likely due to interference from binding globulins. Limited availability of experimental animals precluded our development of a reliable ACTH assay in this species. We therefore tested the effects of exogenous ACTH and found that midday intramuscular injections of ACTH (30 nM/g) rapidly increased EOD amplitude by 38.7%±5.3% SEM (Figure 4A and 4B), while saline injections at this time point had little or no effect, increasing amplitude by only 8.4%±1.5%. Injection of ACTH also caused a small but reliable increase in the EOD pulse width (Figure 4C). EOD rate, regulated by pacemaker neurons in the medulla, was unaffected by saline or ACTH injections (*t* = 0.54, df = 14, *p*>0.5).

### Melanocortin Peptides Increase Na^+^ and Inward Rectifying K+ Currents

We next evaluated the effects of ACTH on the electrocyte AP in vitro. APs were recorded in normal saline and at 5 min intervals following bath application of 100 nM ACTH or saline. After 20 min of exposure to ACTH, AP amplitude increased by 24.5%±5.3% while AP amplitude in saline controls declined by 2.16%±2.5% (Figure 4D and 4E). The effects of ACTH on AP amplitude were rapid—ACTH increased AP amplitude measurably after only 5 min (Figure 4F). ACTH also increased AP duration by 20.3%±7.2%, while saline exposure increased AP duration by only 3.9%±3.2% (Figure 4E). Input resistance in ACTH-treated cells did not change relative to saline controls (*t* = 0.18, df = 9, *p* = 0.86).

Electrocytes of *S. macrurus* have three voltage-gated ion currents: an inactivating Tetrodotoxin (TTX)-sensitive Na^+^ current (INa), a classical delayed rectifier potassium current (IK_DR_), and an inward rectifier potassium current (IK_IR_) [8]. We used two-electrode voltage clamp to further analyze how ACTH increases AP (and thereby EOD) amplitude. Changes in AP amplitude in this species are due to changes in the properties of INa. IK_DR_ activates with a time-constant of 4.6±0.3 ms [16], too slowly to play a role in shaping the AP upstroke or peak amplitude and, more conclusively, we found that AP amplitude is not affected by blocking IK_DR_ with 60 mM tetraethylammonium (TEA). AP amplitude in seven cells was the same at baseline (97.0±10.6 mV) and after 15 min treatment with TEA (99.2±16.8 mV; *t* = 0.75, df = 6, *p* = 0.48).

**Figure 5:**
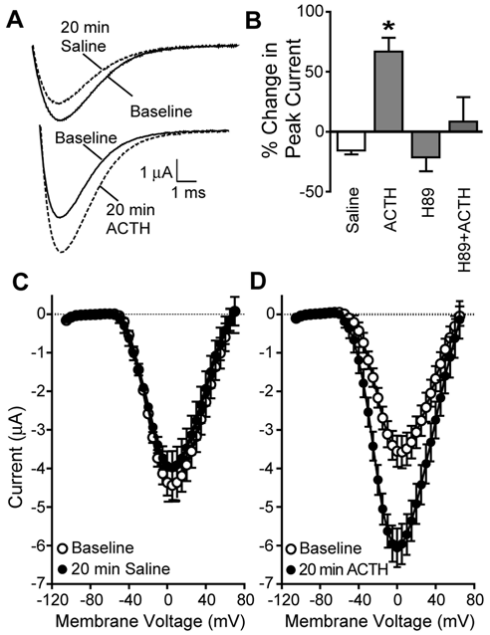
ACTH increases INa magnitude via the cAMP/PKA pathway. (A) Sodium currents recorded before and after 20 min exposure to saline or ACTH. Sodium currents were pharmacologically isolated by blocking potassium currents with 60 mM TEA. (B) Sodium current increases after 20 min exposure to ACTH. The PKA blocker H89 has no effect alone, but pretreatment with H89 blocked the ACTH-induced increase in Na^+^ current magnitude (*n* = 5 per condition, ANOVA *F*_[3, 16]_ = 9.348, *p*<0.001). (C) Normalized Na^+^ I-V curves for cells before and after 20 min saline-control treatment. (D) Normalized Na^+^ I-V curves for cells before and after 20 min exposure to ACTH. Voltage of peak INa was unchanged in both control and ACTH-treated cells (paired *t*-test, df = 4, *p*>0.3 for both saline and ACTH).

Treatment with ACTH strongly boosted the Na^+^ current amplitude by 66.6%±11.7% while saline controls decreased it by 15.7%±3.3% (Figure 5). The effect of ACTH on INa amplitude was as rapid as for the whole cell AP, with marked amplitude increase apparent after 5 min, the first time point sampled. In contrast, ACTH had no effect on voltage dependence (Figure 5) or inactivation kinetics of INa; percentage change in inactivation tau after 20 min was −5.4%±3.0% in control cells and 0.0%±2.5% in ACTH-treated cells, a nonsignificant difference (*t* = 1.79, df = 18, *p* = 0.10).

We then investigated the signal transduction pathway by which ACTH increases the magnitude of INa. Melanocortin peptides, including ACTH, initiate their actions via membrane-bound melanocortin receptors, all of which are coupled positively to adenylate cyclase and elevate intracellular cAMP when activated in other systems [17]. We found previously that the membrane-permeant cAMP analog 8-Br-cAMP increases EOD, AP, and INa amplitude by activating of the PKA pathway [14],[18]. Thus, melanocortins released from the pituitary likely serve as the signal from the brain to the periphery that initiates the cAMP cascade that regulates electrocyte INa in *S. macrurus*. To test this hypothesis we pretreated cells with 30 µM H89, a cell permeable PKA inhibitor that blocks PKA activation, and then added ACTH (100 nM). H89 pretreatment blocked the ability of ACTH to boost INa amplitude, indicating that the observed ACTH effects on electrocyte INa (and thereby AP amplitude) are mediated via PKA (Figure 5B).

**Figure 6:**
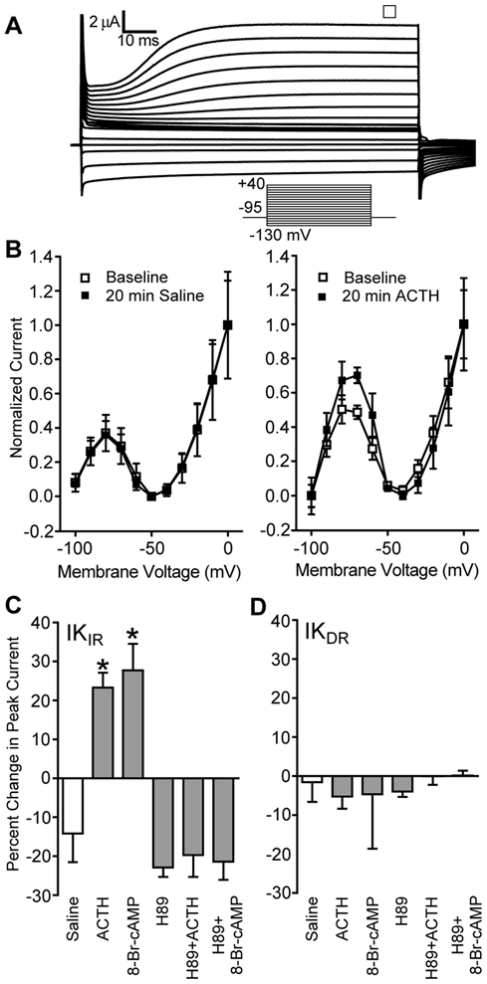
ACTH increases the inward rectifier potassium current (IK_IR_) via a cAMP/PKA pathway; the delayed rectifier current (IK_DR_) is stable across all conditions. (A) Representative family of electrocyte potassium currents recorded with INa blocked by 1 µM TTX. To generate the IV curves in Panel B, current amplitude was measured at steady state (open square in A). (B) Normalized IV curves at baseline and after 20 min exposure to saline (control) or ACTH. (C) Both ACTH and the cAMP analog 8-Br-cAMP increase the magnitude of IK_IR_. The PKA blocker H89 had no effect alone, but pretreatment with H89 blocked the ACTH- and 8-Br-cAMP-induced increase in IK_IR_ magnitude. Asterisks indicate conditions different from saline controls (ANOVA *F*_[5, 34]_ = 16.22, *p*<0.0001; pairwise comparisons by Tukey's HSD). (D) The delayed rectifier was stable across all experimental conditions (ANOVA *F*_[5, 34]_ <1, *p*>0.5).

Neither ACTH (100 nM) nor 8-Br-cAMP (1 mM) affected the delayed rectifier (Figure 6D), which is the outward current seen at −30 mV to 0 mV on the IV curves (Figure 6B). However, ACTH and 8-Br-cAMP increased the peak amplitude of the inward rectifier at −80 mV and −90 mV (Figure 6B and 6C). The effects of ACTH and of 8-Br-cAMP were blocked by H89 (30 µM), verifying that they act via PKA.

No treatment-related effects were seen on resistance, threshold of activation for any of the three currents, membrane voltage of peak INa or peak IK_IR_, recovery from inactivation of INa, steady state inactivation of INa, inactivation time-constant of INa, or activation time-constant of IK_DR_.

It was hypothesized previously that increasing IK_IR_ decreases resting membrane resistance and the internal resistance of the electric organ such that each EOD produces a stronger electric field in the surrounding water [14]. However, because IK_IR_ is active below the threshold for INa activation and AP initiation, it now seems unlikely that changes in IK_IR_ affect the AP waveform. Rather our voltage-clamp data confirm that ACTH-induced increases in AP amplitude and duration result entirely from increases in INa magnitude, a change mediated by the cAMP/PKA pathway.

### Rapid Increases in EOD Amplitude Are Due to Hormonal Enhancement of Constitutive Trafficking of Na^+^ Channels

Because PKA induces no changes in kinetic or voltage-dependent properties of INa [19] and IK_IR_ (unpublished data), we hypothesized that melanocortins increase INa and IK_IR_ by increasing the number of channels active in the cell membrane. One mechanism would be to increase trafficking of preformed channels from a vesicular reservoir into the plasma membrane. To evaluate this hypothesis we applied compounds that disrupt ion channel processing and insertion at different stages in the protein synthesis-exocytosis pathway. Chloroquine (CQ) interferes with vesicle recycling [20], membrane fusion events [21], and Na^+^ channel trafficking [10],[22],[23]. N-ethylmaleimide (NEM) is widely used to disrupt vesicle docking and recycling. In contrast to CQ and NEM, which primarily disrupt vesicle trafficking at the plasma membrane, Brefeldin A (BFA) interferes with earlier protein processing by disrupting transport of proteins from the endoplasmic reticulum to the Golgi apparatus and also slows the recycling of endocytosed proteins [24].

**Figure 7:**
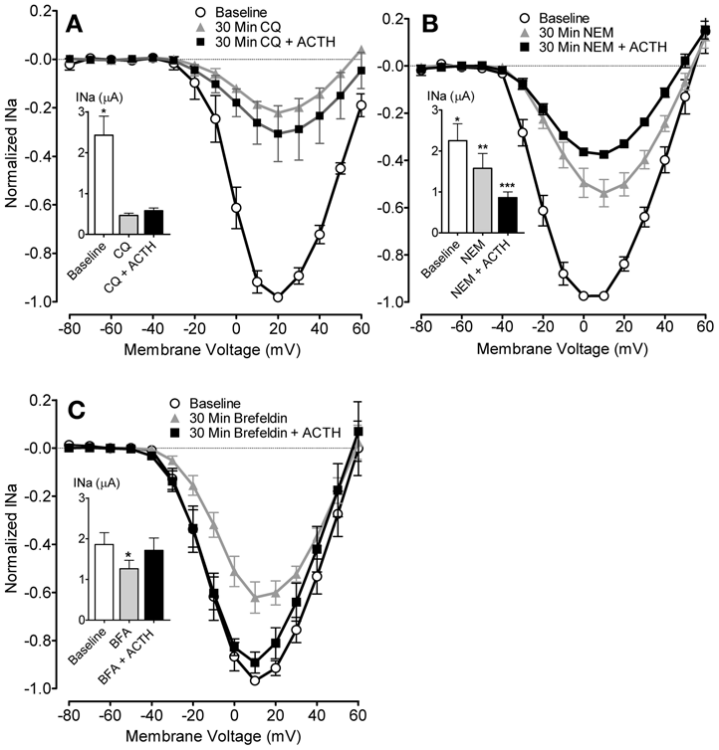
Disrupting vesicular trafficking prevents the ACTH-induced increase in INa. (A–C) CQ and NEM prevented the ACTH-induced increase in INa magnitude, whereas BFA did not prevent the ACTH-induced current enhancement. Potassium currents were blocked in all conditions with 60 mM TEA. I-V curves depict normalized INa at baseline and after 30 min exposure to CQ, NEM, or BFA, then 30 min after addition of ACTH. Insets: summary of peak INa at baseline and after 30 min exposure to CQ, NEM, or BFA, then 30 min after addition of ACTH. Asterisks indicate conditions significantly different from other conditions by Tukey's HSD following significant omnibus repeated measures ANOVA (for CQ *F*_[2,4,8]_ = 17.13, *p*<0.01; NEM *F*_[2,4,8]_ = 17.28, *p*<0.01; BFA *F*_[2,5,10]_ = 16.16, *p*<0.001).

We pretreated electrocytes for 30 min with either 50 µM CQ, 100 µM NEM, or 150 µM BFA, followed by the addition of 100 nM ACTH for 30 min. All three compounds reduced INa amplitude (Figure 7A–7C), with CQ causing an especially pronounced reduction in INa (to 29.09%±1.07% of baseline). Both CQ and NEM blocked the effects of ACTH (Figure 7). In contrast, BFA did not block the ACTH-induced increase in INa amplitude (Figure 7), indicating that ACTH works downstream of protein processing, so the mechanism of ACTH action cannot be synthesis of new Na^+^ channels.

Reduction of INa by CQ and NEM could result from interference with the trafficking of either Na^+^ channels or melanocortin receptors. We discriminated between these possibilities by pretreating cells with CQ or NEM before activating PKA directly with 8-Br-cAMP. Both CQ (*n* = 4) and NEM (*n* = 4) prevented the PKA-induced INa increase (unpublished data), supporting our conclusion that these compounds act downstream of the ACTH receptor, i.e., on Na^+^ channels.

**Figure 8:**
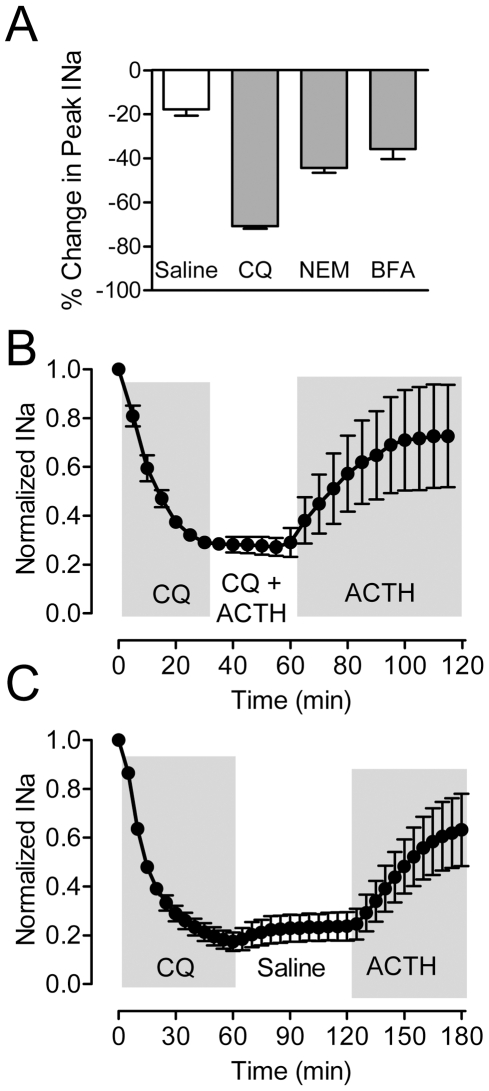
Rundown of sodium current and washout of CQ effect. (A) Decrease in peak Na^+^ current following 30 min exposure to Saline, CQ, NEM, and BFA. (B) Washing out CQ while maintaining ACTH in the bath saline led to increased INa magnitude (*n* = 4). (C) Washing out CQ with normal saline for 60 min did not produce recovery of INa, but subsequent addition of ACTH increased INa magnitude (*n* = 4).

Control cells in normal saline show a gradual decrease in INa [19] and we observed similar rundown in the present experiments (Figures 5B and 8A). This rundown was increased by CQ, NEM, and BFA (Figure 8A), with CQ causing the largest decrease in peak current. Given the extreme and rapid attenuation of INa magnitude by CQ, we tested the capacity of cells to respond to ACTH after CQ washout. After pretreatment with CQ, we exposed cells to CQ and ACTH for 30 min, followed by 60 min treatment with ACTH alone (Figure 8B). During the final ACTH treatment, INa increased over the course of 60 min, indicating that the effects of CQ are reversible. However, the increase in INa during ACTH treatment could have resulted either from CQ washout or the combined washout of CQ and the presence of ACTH. We therefore tested whether the increased INa during ACTH treatment resulted only from the washout of CQ. Four cells were exposed first to CQ for 60 min, then a 60 min saline washout, followed by a 60 min application of ACTH. CQ reduced INa to 17.4%±3.7% of its baseline magnitude after 60 min (Figure 8C). After 60 min of saline washout, INa magnitude recovered only to 23.7%±5.8% of baseline, a nonsignificant recovery. In contrast, after 60 min of ACTH exposure, INa recovered to 63.3%±14.8% of its baseline value, indicating that washout of CQ alone is not sufficient for recovery of INa, and that the presence of ACTH is necessary for this recovery.

**Figure 9:**
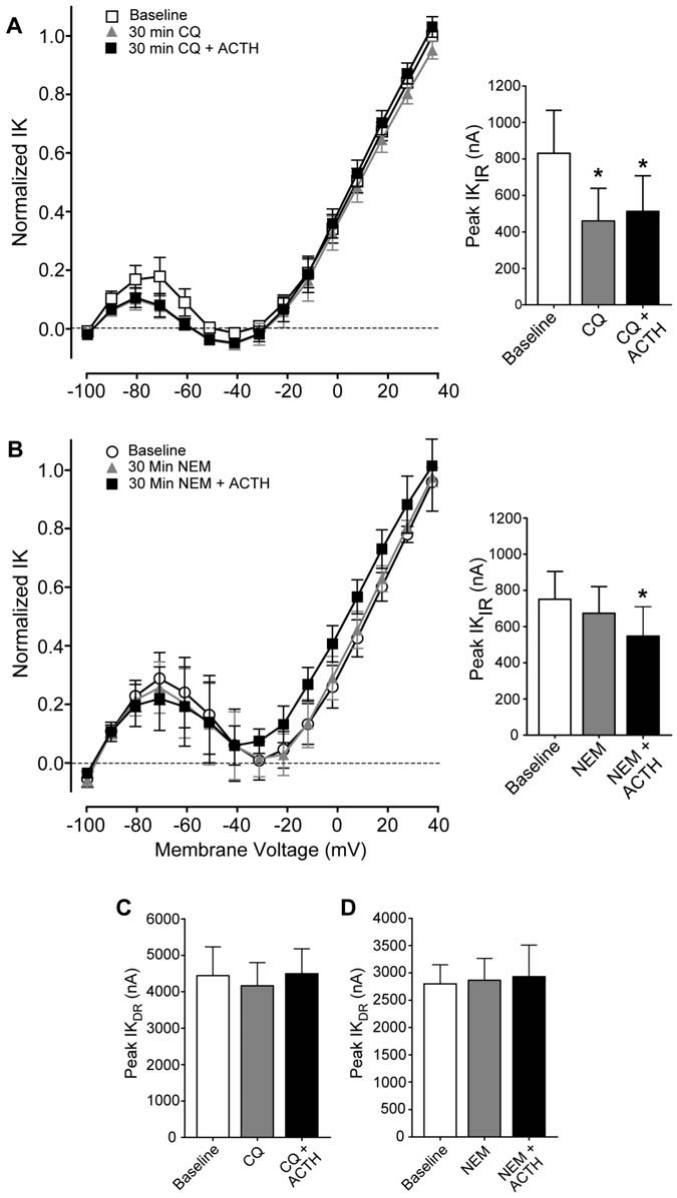
Disrupting vesicular trafficking prevents the ACTH-induced increase in the inward rectifier potassium current (IK_IR_) but has no effect on the delayed rectifier current (IK_DR_). (A,B) CQ and NEM prevented the ACTH-induced increase in IK_IR_ magnitude. I-V curves depict normalized steady-state IK at baseline and after 30 min exposure to CQ or NEM, then 30 minutes after addition of ACTH. Potassium currents are isolated in all conditions by blocking INa with 1 µM TTX. The apparent increase in DR current with NEM treatment plus ACTH is not statistically significant when compared at individual membrane voltages. Insets: summary of peak IK_IR_ at baseline and after 30 min exposure to CQ or NEM, then 30 min after addition of ACTH. Both compounds produced a decrease in current magnitude. Asterisks indicate conditions significantly different from baseline by Tukey's HSD following significant omnibus repeated measures ANOVA (for CQ *F*_[2,4,8]_ = 26.6, *p*<0.001; NEM *F*_[2,5,10]_ = 6.00, *p*<0.05). (C) The magnitude of IK_DR_ at peak current is not changed by CQ or CQ with ACTH (*F*_[2,4,8]_ = 2.76, *p*>0.1). (D) The magnitude of IK_DR_ at peak current was not changed in the presence of NEM or NEM with ACTH (*F*_[2,5,10]_ = 0.12, *p*>0.8).

Neither CQ nor NEM inhibited IK_DR_ (Figure 9C and 9D), providing strong evidence that these compounds do not cause a nonspecific decline in cell function. CQ pretreatment reduced peak IK_IR_, while NEM did not significantly inhibit IK_IR_ (Figure 9B). Both compounds blocked the ACTH-induced enhancement of IK_IR_ (Figure 9A and 9B), suggesting that ACTH also enhances IK_IR_ by up-regulating vesicular trafficking and that CQ and NEM block the effects of ACTH by inhibiting this process.

**Figure 10:**
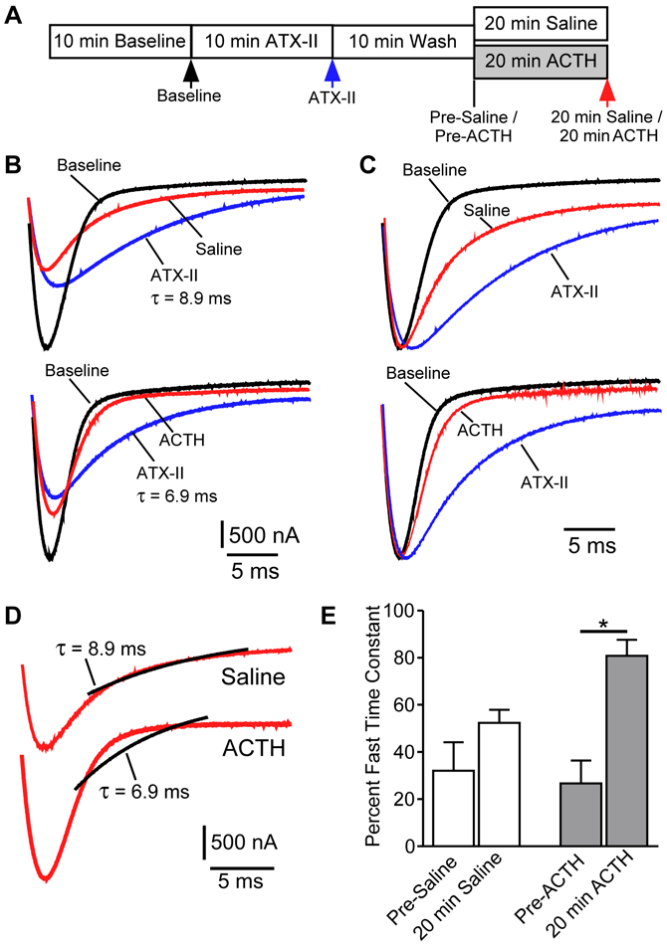
ACTH promotes recovery of fast INa inactivation following ATX-II treatment. (A) Schematic experimental timeline indicating points of INa measurement represented in Panels B–E. Color-coded arrowheads correspond to the colors of representative traces shown in B–D. (B) Representative peak magnitude INa traces at baseline, after ATX-II saturation, and after washout of ATX-II followed by 20 min exposure to saline (top) or ACTH (bottom). Potassium currents were blocked in all conditions by addition of 60 mM TEA. (C) Same traces as in (B), but current amplitudes are normalized to baseline to facilitate direct comparison of the inactivation phase in each current. (D) Current traces following 20 min treatment with saline or ACTH treatment taken from Panel B. Black lines represent the best possible single exponential fit using the slow inactivation time-constant from the corresponding ATX-treated traces. The slow time-constant better accounts for inactivation in the saline treated cell. (E) After washout of ATX-II, τ_fast_ accounts for approximately 25% of INa inactivation in all cells prior to 20 min exposure to saline or ACTH. After the 20 min experimental manipulation, the fast component of INa inactivation shows greater recovery in cells treated with ACTH than in saline controls (*F*_[3,12]_ = 7.612, *p*<0.01; pairwise comparison significantly different *p*<0.01 by Tukey's HSD).

In a final experiment, we supplemented our pharmacological data supporting ACTH-induced channel trafficking with additional evidence that ACTH leads to the insertion of new Na^+^ channels into the membrane. We took advantage of the fact that electrocyte INa normally inactivates with a single time-constant (τ_fast_) (Figure 10). We applied a high-affinity sea-anemone toxin (ATX-II), which binds to extracellular loops [25] of voltage-gated Na^+^ channels in the membrane, slowing INa inactivation. After exposure to saturating concentrations of ATX-II, electrocyte INa inactivated with a single slower time-constant (τ_slow_) (Figure 10). Following rapid washout of unbound ATX-II from the bath, INa inactivated with a dual time-constant, reflecting the relative contributions of channels with and without bound toxin. Immediately after ATX-II washout, approximately 25% of INa inactivation was accounted for by τ_fast_ when τ_slow_ is constrained to the value derived by single-exponential fit following ATX-II saturation. After additional exposure to normal saline or saline with ACTH for 20 min, τ_fast_ accounted for 52.4%±10.9% of INa inactivation in saline controls, whereas τ_fast_ accounted for 80.8%±13.6% of INa inactivation in ACTH-treated cells (Figure 10). The increased recovery of τ_fast_ in ACTH-treated cells strongly suggests the presence of new channels without bound toxin and supports the conclusion that ACTH treatment works by increasing the rate of channel insertion into the membrane, rather than by slowing endocytosis of membrane-bound channels.

### Increase in Na^+^ Channel Number Is Metabolically Costly

Our experiments establish that *S. macrurus* increases its EOD amplitude to a greater extent than the gymnotiform pulse fish *B. pinnicaudatus*, and that boosting the EOD results entirely from increases in the number of Na^+^ channels present in the electrocyte membrane. Given the potential role of EOD amplitude modulation in regulating energy consumption by the electric organ, we estimated the metabolic cost of increased EOD amplitude by measuring the increase in total Na^+^ influx during electrocyte AP modulations. One molecule of ATP must be expended to pump three Na^+^ ions out of the cell; therefore any increase in Na^+^ movement into the cell will cause a proportional increase in energetic use by the Na^+^/K^+^ ATPase. We considered only changes in Na^+^ influx because the inward rectifier current inactivates well below AP threshold and the delayed rectifier activates too slowly to affect AP waveform [8].

**Figure 11:**
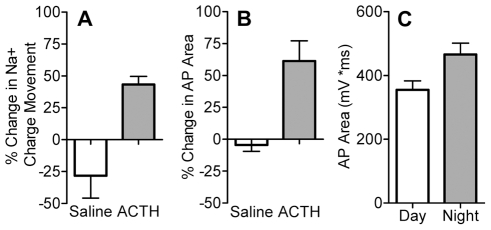
Increased Na^+^ influx during the enhanced electrocyte AP. (A) Total Na^+^ charge movement during peak INa recorded in voltage clamp increases by 43%±6% after treatment with ACTH but decreases by 28%±17% in control cells (*t* = 4.68, df = 9, *p*<0.01). (B) Treatment with ACTH increases area under the AP by 61%±16%, compared to a 5%±5% decrease in controls (*t* = 4.20, df = 13, *p*<0.001). (C) Area under the AP is greater in electrocytes harvested at night than in day-harvested electrocytes (*t* = 2.55, df = 10, *p*<0.05).

Total Na^+^ charge movement, measured as the time integral of the peak Na^+^ current in voltage clamp, increased by 43%±6% after ACTH treatment and decreased by 28%±17% in controls, a difference of 71% (Figure 11A). We also computed the area under the AP as an alternate estimate of Na^+^ charge movement during the AP. Treatment with ACTH led to a 61%±16% increase in AP area, compared to a decrease of 4.5%±5% in saline controls (Figure 11B), a difference of 65%. These values likely represent the lower limit of increased Na^+^ movement and energy demand by the electrocyte—trials with larger *S. macrurus* in another study (P. Stoddard, unpublished data) show that melanocortins can increase total EOD area by as much as 340%, indicating a significant potential for energy expenditure or conservation by dynamic regulation of EOD amplitude.

We estimated increased Na^+^ charge movement under physiological conditions by comparing the time integral of the AP waveform in day- versus night-harvested electrocytes. Electrocyte AP area increased from 355±67 mV*ms during the day to 466±87 mV*ms at night, an increase of 31.3% (Figure 11C). Even by this conservative estimate based on measurements in electrocytes removed from circulating melanocortin peptides, the nighttime increase in EOD amplitude would require a 31% increase in ATP expenditure on active transport to remove the accumulated Na^+^ from the electrocyte.

## Discussion

**Figure 12:**
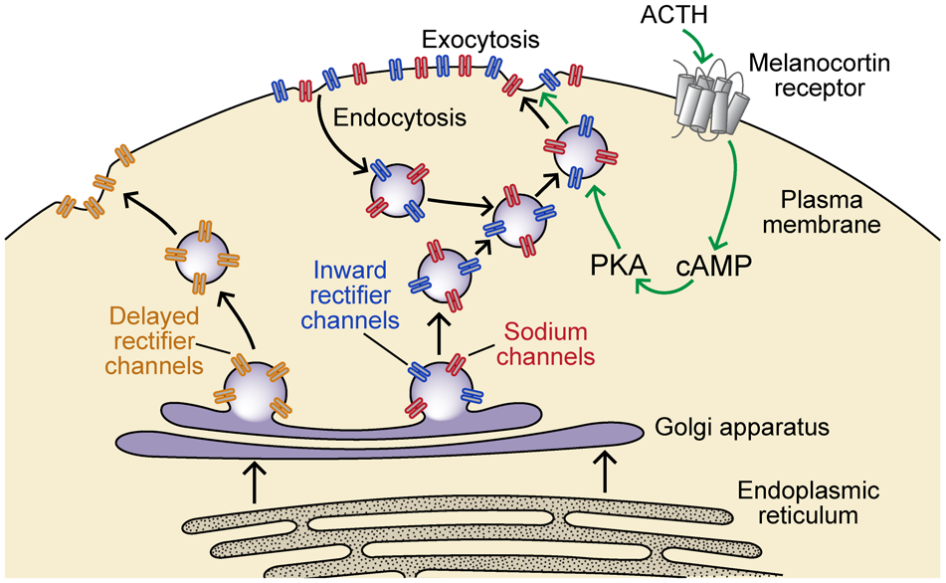
Mechanisms controlling ion channel trafficking in *S. macrurus* electrocytes. Ion channel proteins are synthesized in the endoplasmic reticulum and then further processed and inserted into vesicles in the Golgi apparatus. Delayed rectifier potassium channels undergo exocytosis to the cell surface without subsequent endocytosis. Inward rectifier channels and Na^+^ channels are constitutively cycled into and out of the membrane. This process is accelerated when the melanocortin peptide hormone ACTH activates a G-protein coupled melanocortin receptor. The receptor initiates a signaling cascade that elevates cAMP and activates PKA. PKA then upregulates the exocytosis of channels into the membrane increasing the number of Na^+^ and inward rectifier channels present in the electrocyte membrane, thereby increasing the magnitude of both conductances. For simplicity we have illustrated Na^+^ and inward rectifier channels as being in the same vesicles, although we do not know whether they are in the same or different vesicles.

The EOD amplitude of the wave-type electric fish *S. macrurus* varies with a diurnal rhythm and in response to social encounters. We found that changes in electrocyte AP amplitude underlie the diurnal enhancements of EOD amplitude seen in vivo, and that the melanocortin peptide ACTH increases EOD amplitude in vivo and electrocyte AP amplitude in vitro. This pattern of EOD amplitude enhancement parallels that observed in pulse-type fish but occurs on a larger scale, consistent with our reasoning that wave fish stand to conserve more energy by EOD amplitude modulation than pulse fish. Our most important results show that ACTH activates the cAMP/PKA pathway within the electrocyte to up-regulate two distinct ionic currents, a Na^+^ current and an inward rectifier K^+^ current, by inducing trafficking of channel proteins into the electrocyte membrane. The delayed rectifier potassium current, on the other hand, is not regulated by this mechanism (Figure 12).

Control of cell surface channel number through regulated exocytosis occurs most commonly in situations where channels are constitutively cycled into and out of the plasma membrane [9]. When the rates of exocytosis and endocytosis are in equilibrium the number of surface proteins remains relatively constant. Increasing the rate of exocytosis relative to the rate of endocytosis will increase the number of surface proteins while decreasing the relative rate of exocytosis will reduce the number of surface proteins. Our data suggest that both of the melanocortin-regulated ion channels (INa and IK_IR_) are constitutively cycled into and out of the plasma membrane. Both currents show steady rundown in normal saline. We believe that this rundown results from decreased rates of exocytosis after the electrocytes are removed from circulating endogenous melanocortin hormones. The resulting gradual decrease in residual PKA activity would slow the rate of exocytosis relative to endocytosis, thus decreasing the presence of Na^+^ and inward rectifier channels in the plasma membrane. This rundown is accelerated by compounds that block exocytosis of new channels into the electrocyte membrane independent of residual PKA activity, further decreasing the rate of exocytosis relative to endocytosis.

Constitutive cycling of ion channels is energetically costly, as both endocytosis and exocytosis require hydrolysis of ATP or GTP. The principal advantage of constitutive channel cycling is speed: by altering the rate of endocytosis or exocytosis the number of channels present in the membrane can be rapidly changed without delays associated with synthesizing channel proteins de novo. Moreover, the energetic costs of constitutive channel cycling are minimal compared to the energy used by the Na^+^/K^+^ ATPase to remove Na^+^ that enters the cell during APs [26]. Electrocytes in *S. macrurus* fire round-the-clock at rates of 70–150 Hz, with whole-cell Na^+^ currents of several µA [8] placing extremely high demand on ATP-dependent ion transporters. We estimate that energetic demands of the EOD are increased by at least 30% during periods of EOD amplitude enhancement, and by as much as 340%. Accordingly, *S. macrurus* stands to conserve more energy through reductions in EOD amplitude during periods of inactivity than is expended by the constitutive cycling of channels in the electrocyte. The costs associated with constitutive cycling are therefore a small price to pay for the energetic savings associated with circadian reductions in EOD amplitude and the ability to quickly increase EOD amplitude in response to environmental events.

While increasing electrocyte INa has a clear role in boosting EOD amplitude, the function of dynamically regulating IK_IR_ is less obvious. One possibility is that the increased Na^+^ influx leads to enhanced activity of Na^+^/K^+^ ATPase, accelerating active transport of K^+^ into the electrocyte. In this circumstance, upregulation of the inward rectifier conductance, a tonic K^+^ conductance active at resting potential, would facilitate passive diffusion of excess K^+^ out of the electrocyte.

Cardiac myocytes, which also must maintain a steady rate of APs, express Na^+^ channels (NaV1.5) that undergo constitutive cycling, regulated by G-protein coupled receptors and cAMP/PKA. Early findings that cardiac Na^+^ current is increased by β-endorphin [27] and a G-protein/PKA pathway [28] were followed by the discovery that PKA upregulates trafficking of NaV1.5 into the plasma membrane [22],[29]. This regulation requires the presence of phosphorylation sites and endoplasmic reticulum retention motifs on an intracellular loop of the channel protein [30]. The similar regulation of Na^+^ channel trafficking in cardiac myocytes and electrocytes raises the possibility that regulated constitutive cycling is a general feature of excitable membranes that must maintain constant activity while retaining flexibility in their pattern or extent of excitability.

A novel finding of the present study is the PKA-regulated trafficking of both INa and IK_IR_ in electrocytes. An important question for further investigation is what mechanisms are involved in co-regulating the trafficking of two molecularly distinct ion channels within the cell. Data on the trafficking of cardiac Na^+^ channels suggest that phosphorylation of the channel proteins could be responsible for increased channel exocytosis in electrocytes. However, phosphorylation of vesicle-associated proteins [31] and the SNARE complex proteins [32] also can change the rates of vesicle docking and exocytosis. We do not yet know which phosphorylation events regulate channel trafficking in electrocytes. It is possible that regulation of Na^+^ channel and inward rectifier channel trafficking are modulated by different phosphorylation events, or a single phosphorylation event could control the fate of both channel types. Critical for addressing these questions will be experiments to determine the functional phosphorylation sites, and the subcellular localization of Na^+^ and inward rectifier channels. Sodium and inward rectifier channels could be loaded into the same vesicles, such that upregulating exocytosis of this single pathway inserts both channels into the membrane. Alternatively Na^+^ and inward rectifier channels could be loaded into separate vesicles, and PKA then independently increases the exocytosis rate of both vesicle populations.

Taken together, our results show that *S. macrurus* maintains a circadian rhythm in EOD amplitude and responds to environmental events in a matter of minutes by increasing EOD amplitude. This modulation of an ongoing behavior is a direct function of the rapid trafficking of ion channels, demonstrating in a vertebrate system a clear relationship between ion channel trafficking and behavior. Such “behavior to molecules to behavior” accounts have been worked out in a small number of invertebrate preparations such as *Drosophila* [33], *C. elegans* [34], and *Aplysia* [35], but similar successes with vertebrate behavior have proven more difficult [c.f., 36]. The experiments in this study demonstrate how an electric fish tracks behaviorally relevant environmental cues and regulates ion channel trafficking in real time to boost power of the electric signal at times of highest social activity. Power of the EOD waveform is perfectly proportional to energy consumed by electrolocation, and electric signals use a significant fraction of the energy budget [1]. Thus the ability to restrict EOD intensification to periods of activity and times of social encounters can significantly reduce overall energetic expenditure on communication.

## Materials and Methods

### Animals

Fish were wild caught *Sternopygus macrurus* (gold-lined knife fish) from tropical South America, obtained from Segrest Farms (Gibsonton, FL, USA) and ranging in size from 20 to 30 cm. After acclimation to lab temperature and aquarium water, the fish were housed in communal 300-l tanks at 28°C±1°C with water conductivity of 600–800 µS/cm. At least 4 wk before they were used in experiments, fish were removed from the communal tanks and housed individually in 20-l aquaria.

All methods were approved by the Institutional Animal Care and Use Committees of Florida International University and The University of Texas, and complied with the guidelines given in the Public Health Service Guide for the Care and Use of Laboratory Animals.

### Solutions and Reagents

We obtained all reagents from Sigma (St. Louis, MO, USA), except for TTX, which was purchased from Biomol (Plymouth Meeting, PA, USA), and the sea anemone toxin ATX-II, which was purchased from Alomone Labs (Jerusalem, Israel). The normal saline for in vivo injections and in vitro physiology contained (in mM): 114 NaCl, 2 KCl, 4 CaCl_2_•2H_2_0, 2 MgCl_2_•6H_2_0, 2 HEPES, 6 glucose; pH to 7.2 with NaOH. Some experimental conditions required modifications to the normal saline, detailed below where appropriate.

### EOD Recordings and Injections In Vivo

Our automated system for recording calibrated EODs from freely swimming fish and procedures for injecting fish are described in detail elsewhere [37]. The essential features of this system are a 300-l aquarium divided into three sections, with the outer two sections joined by an unglazed ceramic tube (Figure 2A). Fish in the tank can swim between the two outer sections only by passing through the central tube. Short (2 cm) nichrome electrodes attached to the tube are connected to custom-developed circuitry that detects when the fish is centered within the tube. EODs are amplified and digitized from a different pair of longer (10 cm) nichrome recording electrodes at opposite ends of the tank only when the fish-detecting circuitry signals that the fish is centered in the ceramic tube equidistant from the recording electrodes. For in vivo EOD recordings, fish were placed in the automated measurement tank, which is located in a light- and temperature-controlled room on a 12L∶12D light cycle. We recorded EODs round the clock at intervals of ∼1 min for several days as to assess day-night variation in EOD waveform. Social challenges were accomplished by introducing a second fish into the central compartment of the recording aquarium for 1 h at midday (see Figure 2). The fish could interact electrically and chemically, but not physically.

Injections of ACTH were administered after at least 2 d of baseline EOD recordings. We injected fish (1 µl/g intramuscular) at midday with 30 µM ACTH in normal saline, or with normal saline as a control condition. Each fish served as its own control so a second injection was given the next day. Four fish received ACTH injections followed by saline injection and four fish received the opposite order of injections. The order of saline versus ACTH injection had no effect on the response to ACTH injection (*t* = 0.13, df = 6, *p*>0.8) or saline injection (*t* = 0.23, df = 6, *p*>0.8).

### Electrophysiology

Procedures for recording from the electric organ have been described previously [14],[18]. In brief, we harvested a 2.5–3.0 cm section of the tail, removed the overlying skin, and pinned the exposed electric organ in a Sylgard recording dish containing normal saline with curare (5 mg/l) added to prevent spontaneous contractions from any tail muscles that might be present in the section. Temperature of the preparation was stable at room temperature (24°C±1°C) or maintained at 24.5°C±0.5°C using a TC2-bip bipolar temperature controller with HI–55D heater under the recording dish and HPRE2 Pre–heater (Cell MicroControls, Virginia Beach, VA, USA).

Electrophysiological recordings were made with the Axoclamp 2B or Axoclamp 900 amplifier, controlled by a Digidata 1320A or 1440 DMA interface using pCLAMP 8 or pCLAMP 10 software (Molecular Devices, Sunnyvale, CA, USA). Sampling rate was at least 20 kHz for all experiments. A two-electrode configuration was used in both current-clamp and voltage-clamp experiments. Microelectrodes were pulled from thin-wall borosilicate glass and had resistances of 0.9–1.2 MΩ when filled with 3 M KCl. The bath ground consisted of a chlorided silver wire inserted into a 3 M KCl agar bridge. To depolarize the cell for recording APs, current steps were delivered through a X100 headstage (Axon HS2A, X100 MGU) and membrane voltage recorded through a X1 headstage (HS-2 X1L). In voltage-clamp mode, current was injected using a X10 headstage. Electrodes were placed within the region of the electrocyte's voltage-gated ion channels, the most posterior portion of the cell with a grounded shield placed between the electrodes to prevent capacitive coupling. To improve the space clamp in some voltage-clamp experiments, NaCl in the saline was replaced with sodium methylsulfate at 93.2 mM. The pH was adjusted to 7.2 with 1 M NaOH, bringing the Na^+^ concentration to ∼114 mM. In these cases, baseline recordings for amplitude comparisons were taken after the switch to the sodium methylsulfate saline and all drugs were bath applied in the methylsulfate saline.

For current clamp experiments, we delivered 25 ms current steps from −200 nA to 2000 nA in 100 nA intervals. In some large cells for which this protocol was not sufficient to elicit APs, we delivered current steps from −400 to 4,000 nA in 200 nA intervals to elicit APs. The current clamp protocol was repeated at 5 min intervals. Baseline recordings were made in normal saline before superfusing with additional normal saline as a control, or with a test solution of 100 nM ACTH dissolved in normal saline. We calculated input resistance from the steady-state voltage responses to 200 nA hyperpolarizing and depolarizing currents steps.

In voltage-clamp experiments, we held the cells at resting potential (−85 to −95 mV) and stepped the potential from −110 to +60 mV in 5 or 10 mV increments, although exceptionally large currents in some cells necessitated reducing the number of depolarizing steps to avoid saturating the amplifier. Sodium currents (INa) were isolated by adding 50 mM TEA to the saline, and potassium currents were isolated by addition of 1 µM TTX. In the presence of 1 µM TTX, the electrocyte displays only a classical delayed rectifier potassium current (IK_DR_), and an inward rectifier potassium current (IK_IR_). These potassium currents can be separated based on different gating properties. The inward rectifier is open at voltages more hyperpolarized than −50 mV and inactivated above this membrane potential, while IK_DR_ activates above −40 mV [8].

In all voltage-clamp experiments, test compounds were dissolved in the recording saline and bath-perfused into the recording chamber. For experiments where we recorded INa in the presence of BFA, CQ, or NEM, we compensated for the addition of 50 mM TEA with an equimolar reduction in Na^+^ to prevent osmotic stress on the cells and facilitate voltage clamp of the Na^+^ currents.

### Data Treatment

EODs recorded in vivo were analyzed using custom software developed in MATLAB (Mathworks, Natick, MA, USA). Amplitude of the EOD was measured peak-to-peak and EOD half-width was measured as the pulse duration at 50% of peak amplitude. Current clamp data were analyzed with MATLAB. AP amplitude was measured from the membrane potential at AP peak to the minimum membrane potential between the AP peak and the end of the current step. We took the maximum AP amplitude measured during each set of current clamp steps. Duration of the AP was measured as the spike width at half amplitude. Input resistance was measured by taking the inverse of the slope from a straight-line fit to the voltage-current plots for the two hyperpolarizing current steps and the first two depolarizing current steps.

Voltage-clamp data were analyzed with Clampfit 8 or 10 (Molecular Devices). For Na^+^ currents, the leak was estimated at the end of the 25 ms test pulse when INa was fully inactivated. In the presence of TEA, in this region of the trace, only a steady state linear leak current remains. The leak was then subtracted from the raw current traces to yield isolated INa traces from which current measurements were made to generate IV curves. All amplitude and inactivation time-constant comparisons were made at the peak Na^+^ current. We estimated inactivation tau by fitting exponential decay functions to the current trace from the first inflection point after the peak in the current curve to the point at which the current declined to 10% of its peak value. In *S. macrurus* electrocytes, INa inactivates with a single time-constant, except during our final experiment when the sea anemone toxin ATX-II introduced a second, slow time-constant. Inactivation tau of INa was therefore estimated by fitting the appropriate region of the trace with a standard single-exponential decay function:
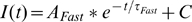
or a two-term standard exponential function:
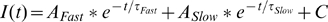
where I(t) is the current at time *t*, A_Fast_ and A_Slow_ are the proportions of current inactivating with the time-constants τ_fast_ and τ_slow_, and C is the non-inactivating component of the current at steady state. For the double-exponential fits, we constrained τ_slow_ to the value derived from the single exponential fit of inactivation following ATX-II saturation.

There are two potassium currents in the electrocyte: a non-inactivating delayed rectifier IK_DR_ that activates starting at −40 mV after a slight delay and an inward rectifier IK_IR_ that is inactivated above −50 mV [8]. Passive leak was estimated from recordings above −40 mV, measuring the region of the trace before activation of the delayed rectifier. The linear leak current was then subtracted from the traces and the IV curves were generated from the leak-corrected traces. Maximum amplitude for IK_IR_ was taken at the peak for this current, which is readily apparent from the IV curves, and IK_DR_ amplitude was measured at 0 mV. Activation tau for IK_DR_ was estimated by fitting the trace with an exponential power function:
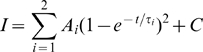



*t*-tests were used when only two groups were compared while comparisons between three or more groups were made using one-way ANOVA with repeated measures. Significant omnibus ANOVAs were further analyzed with post hoc pairwise comparisons using Tukey's or Bonferonni correction to maintain experiment-wise alpha at 0.05. All statistical analyses were performed with MATLAB or Prism 5 (GraphPad, San Diego, CA, USA). Group data are reported as mean±SEM.

## Abbreviations

ACTH: adrenocorticotropic hormone
APs: action potentials
BFA: Brefeldin A
CQ: Chloroquine
EODs: electric organ discharges
NEM: N-ethylmaleimide
TEA: Tetraethylammonium
TTX: Tetrodotoxin

## References

[1] Salazar V. L, Stoddard P. K (2008). Sex differences in energetic costs explain sexual dimorphism in the circadian rhythm modulation of the electrocommunication signal of the gymnotiform fish *Brachyhypopomus pinnicaudatus*.. J Exp Biol.

[2] Hanika S, Kramer B (1999). Electric organ discharges of mormyrid fish as a possible cue for predatory catfish.. Naturwissenschaften.

[3] Stoddard P. K (1999). Predation enhances complexity in the evolution of electric fish signals.. Nature.

[4] Hanika S, Kramer B (2000). Electrosensory prey detection in the African sharptooth catfish, *Clarias gariepinus* (Clariidae), of a weakly electric mormyrid fish, the bulldog (*Marcusenius macrolepidotus*).. Behav Ecol Sociobiol.

[5] Stoddard P. K, Markham M. R, Salazar V. L, Allee S (2007). Circadian rhythms in electric waveform structure and rate in the electric fish *Brachyhypopomus pinnicaudatus*.. Physiol Behav.

[6] Markham M. R, Stoddard P. K (2005). Adrenocorticotropic hormone enhances the masculinity of an electric communication signal by modulating the waveform and timing of action potentials within individual cells.. J Neurosci.

[7] Markham M. R, Allee S. J, Goldina A, Stoddard P. K (2009). Melanocortins regulate the electric waveforms of gymnotiform electric fish.. Horm Behav.

[8] Ferrari M. B, Zakon H. H (1993). Conductances contributing to the action potential of Sternopygus electrocytes.. J Comp Physiol A Neuroethol Sens Neural Behav Physiol.

[9] Royle S. J, Murrell-Lagnado R. D (2003). Constitutive cycling: a general mechanism to regulate cell surface proteins.. Bioessays.

[10] Vijayaragavan K, Boutjdir M, Chahine M (2004). Modulation of Nav1.7 and Nav1.8 peripheral nerve sodium channels by protein kinase A and protein kinase C.. J Neurophysiol.

[11] Faber E. S. L, Delaney A. J, Power J. M, Sedlak P. L, Crane J. W (2008). Modulation of SK channel trafficking by beta adrenoceptors enhances excitatory synaptic transmission and plasticity in the amygdala.. J Neurosci.

[12] Kim J, Jung S. C, Clemens A. M, Petralia R. S, Hoffman D. A (2007). Regulation of dendritic excitability by activity-dependent trafficking of the A-type K+ channel subunit Kv4.2 in hippocampal neurons.. Neuron.

[13] Cusdin F. S, Clare J. J, Jackson A. P (2008). Trafficking and cellular distribution of voltage-gated sodium channels.. Traffic.

[14] McAnelly L, Silva A, Zakon H. H (2003). Cyclic AMP modulates electrical signaling in a weakly electric fish.. J Comp Physiol A Neuroethol Sens Neural Behav Physiol.

[15] Singley J. A, Chavin W (1976). The diel rhythm of circulating ACTH titer in the goldfish (Carassius auratus l.).. Comp Biochem Physiol A Comp Physiol.

[16] McAnelly M. L, Zakon H. H (2007). Androgen modulates the kinetics of the delayed rectifying K(+) current in the electric organ of a weakly electric fish.. Dev Neurobiol.

[17] Hadley M. E, Haskell-Luevano C (1999). The proopiomelanocortin system.. Ann N Y Acad Sci.

[18] McAnelly M. L, Zakon H. H (2000). Coregulation of voltage-dependent kinetics of Na(+) and K(+) currents in electric organ.. J Neurosci.

[19] McAnelly L, Zakon H. H (1996). Protein kinase A activation increases sodium current magnitude in the electric organ of *Sternopygus*.. J Neurosci.

[20] Tixier-Vidal A, Moreau M. F, Picart R, Gourdji D (1982). Effect of chloroquine on thyroliberin interaction with clonal rat prolactin cells. Cytochemical correlates.. Neuroendocrinology.

[21] Doi H, Ishii A, Shimono K (1988). A rapid in vitro assay system using anti-bromodeoxyuridine for drug susceptibility of Plasmodium falciparum.. Trans R Soc Trop Med Hyg.

[22] Zhou J, Yi J, Hu N, George A. L, Murray K. T (2000). Activation of protein kinase a modulates trafficking of the human cardiac sodium channel in xenopus oocytes.. Circ Res.

[23] Chahine M, Ziane R, Vijayaragavan K, Okamura Y (2005). Regulation of Na-v channels in sensory neurons.. Trends Pharmacol Sci.

[24] Jareb M, Banker G (1997). Inhibition of axonal growth by brefeldin A in hippocampal neurons in culture.. J Neurosci.

[25] Rogers J. C, Qu Y, Tanada T. N, Scheuer T, Catterall W. A (1996). Molecular determinants of high affinity binding of alpha -scorpion toxin and sea anemone toxin in the S3–S4 extracellular loop in domain iv of the Na+ channel alpha subunit.. J Biol Chem.

[26] Attwell D, Laughlin S. B (2001). An energy budget for signaling in the grey matter of the brain.. J Cereb Blood Flow Metab.

[27] Lu T, Lee H. C, Kabat J. A, Shibata E. F (1999). Modulation of rat cardiac sodium channel by the stimulatory G protein alpha subunit.. J Physiol.

[28] Matsuda J. J, Lee H, Shibata E. F (1992). Enhancement of rabbit cardiac sodium channels by beta-adrenergic stimulation.. Circ Res.

[29] Hallaq H, Yang Z, Viswanathan P. C, Fukuda K, Shen W (2006). Quantitation of protein kinase A-mediated trafficking of cardiac sodium channels in living cells.. Cardiovasc Res.

[30] Zhou J, Shin H. G, Yi J, Shen W, Williams C. P (2002). Phosphorylation and putative ER retention signals are required for protein kinase A-mediated potentiation of cardiac sodium current.. Circ Res.

[31] Ghosh P, Kornfeld S (2003). AP-1 binding to sorting signals and release from clathrin-coated vesicles is regulated by phosphorylation.. J Cell Biol.

[32] Snyder D. A, Kelly M. L, Woodbury D. J (2006). SNARE complex regulation by phosphorylation.. Cell Biochem Biophys.

[33] Carew T. J, Sahley C. L (1986). Invertebrate learning and memory: from behavior to molecules.. Annu Rev Neurosci.

[34] Rankin C. H (2002). From gene to identified neuron to behaviour in Caenorhabditis elegans.. Nat Rev Genet.

[35] Kandel E. R (2001). The molecular biology of memory storage: a dialogue between genes and synapses.. Science.

[36] Seehausen O, Terai Y, Magalhaes I. S, Carleton K. L, Mrosso H. D (2008). Speciation through sensory drive in cichlid fish.. Nature.

[37] Stoddard P. K, Markham M. R, Salazar V. L (2003). Serotonin modulates the electric waveform of the gymnotiform electric fish *Brachyhypopomus pinnicaudatus*.. J Exp Biol.

[38] Franchina C. R, Stoddard P. K (1998). Plasticity of the electric organ discharge waveform of the electric fish *Brachyhypopomus pinnicaudatus* - I. Quantification of day-night changes.. J Comp Physiol A Neuroethol Sens Neural Behav Physiol.

